# Supplementary material for: The Release of Antimony from Mine Dump Soils in the Presence and Absence of Forest Litter
Source: Int J Environ Res Public Health. 2018 Nov 24;15(12):2631. doi: 10.3390/ijerph15122631 (PMC6313715; doi:10.3390/ijerph15122631)
Supplement: Supplementary file 1 [file ijerph-15-02631-s001.pdf]

Supplementary material

Table S1.
The values of Eh and pH of soil pore water collected after 2, 7, 14, 28 and 90 days [a)] of incubation.

Soil	Treatment	Eh, mV					pH				
		2 d	7 d	14 d	28 d	90 d	2 d	7 d	14 d	28 d	90 d
ZS 1	0/80%	230	150	173	211	225	5.75	6.74	6.47	6.69	6.35
	FL/80%	211	116	153	180	180	5.08	7.58	7.33	7.36	7.42
	0/100%	328	302	217	196	250	7.64	7.78	7.97	7.93	7.75
	FL/100%	147	189	166	199	65	8.21	7.79	8.30	7.89	7.62
ZS 2	0/80%	106	97	103	137	155	8.36	8.43	8.76	8.38	8.37
	FL/80%	91	50	82	123	135	8.51	8.58	8.81	8.42	8.32
	0/100%	158	160	151	160	181	8.35	8.37	8.59	8.59	8.22
	FL/100%	148	170	149	174	38	8.39	8.47	8.57	8.40	8.30
DB	0/80%	306	282	271	261	nd [b)]	3.63	3.86	3.97	4.45	nd
	FL/80%	285	253	229	237	nd	3.81	4.21	4.72	5.53	nd
	0/100%	243	239	330	273	nd	5.39	4.97	4.21	4.76	nd
	FL/100%	241	148	165	209	nd	5.51	5.01	5.58	4.57	nd
DM	0/80%	159	148	144	161	189	7.90	7.92	8.14	8.00	8.12
	FL/80%	182	148	108	137	210	5.44	7.82	8.57	8.26	6.95
	0/100%	205	192	201	168	197	7.99	8.14	8.00	8.03	7.58
	FL/100%	142	174	149	125	110	8.17	7.09	8.12	8.17	6.70
R	0/80%	286	277	295	274	261	5.56	5.96	5.24	5.75	5.62
	FL/80%	263	211	224	257	236	5.47	6.09	5.82	5.71	5.80
	0/100%	274	262	256	245	298	5.31	6.48	6.87	6.82	5.17
	FL/100%	228	223	203	185	130	6.36	6.46	4.37	4.57	6.11
SG	0/80%	199	175	167	186	nd	8.23	8.53	8.52	8.44	nd
	FL/80%	205	188	162	169	nd	8.11	7.65	7.76	7.83	nd
	0/100%	185	150	149	168	180	8.27	8.30	8.41	8.27	8.35
	FL/100%	145	147	127	136	107	7.54	7.30	8.33	7.66	7.98

All the data are mean values of 3 replicates. The results obtained for replicates differed considerably, and related values of standard deviation SD were in the ranges: 9-43 mV for Eh, and 0.02-0.31 for pH.

[a)] The final measurement was delayed for 3 days after acquiring pore water. Collected samples were stored in the fridge (4ºC) prior to the measurement.

[b)] nd – not determined
